# 11C-acetate positron emission tomography is more precise than 18F-fluorodeoxyglucose positron emission tomography in evaluating tumor burden and predicting disease risk of multiple myeloma

**DOI:** 10.1038/s41598-021-01740-2

**Published:** 2021-11-12

**Authors:** Miao Chen, Wenjia Zhu, Jianhua Du, Chen Yang, Bing Han, Daobin Zhou, Li Huo, Junling Zhuang

**Affiliations:** 1 Department of Hematology, Peking Union Medical College Hospital, Peking Union Medical College and Chinese Academy of Medical Sciences, Beijing, China; 2 Department of Nuclear Medicine, Peking Union Medical College Hospital, Peking Union Medical College and Chinese Academy of Medical Sciences, Beijing, China

**Keywords:** Myeloma, Radionuclide imaging, Whole body imaging

## Abstract

The optimal method of tumor burden evaluation in newly diagnosed multiple myeloma (NDMM) is yet to be determined. This study aimed to compare the value of ^11^C-acetate positron-emission tomography (PET)/computed tomography (CT) (AC-PET and ^18^F-fluorodeoxyglucose PET/CT (FDG-PET) in the assessment of tumor burden in NDMM. This study evaluated 64 NDMM patients between February 2015 and July 2018. AC-PET and FDG-PET were used to assess myeloma lesions. The clinical data, imaging results, and their correlations were analyzed. Diffuse bone marrow uptake in AC-PET was significantly correlated with biomarkers for tumor burden, including serum hemoglobin (*P* = 0.020), M protein (*P* = 0.054), the percentage of bone marrow plasma cells (*P* < 0.001), and the Durie–Salmon stage of the disease (*P* = 0.007). The maximum standard uptake value (SUV_max_) of focal lesions and high diffuse bone marrow uptake in AC-PET showed stronger correlations with high-risk disease (*P* = 0.017, *P* = 0.013) than those in FDG-PET. Moreover, the presence of diffuse bone marrow uptake, more than ten focal lesions, and an SUV_max_ of focal lesions of > 6.0 in AC-PET, but not in FDG-PET, predicted a higher probability of disease progression and shorter progression-free survival (*P* < 0.05). AC-PET outperformed FDG-PET in tumor burden evaluation and disease progression prediction in NDMM.

## Introduction

Multiple myeloma (MM), a malignant plasma cell proliferative disorder characterized by bone marrow infiltration and the production of abnormal monoclonal immunoglobulin known as M-protein, can lead to impaired immune function, high blood viscosity, and multiple organ damage, particularly kidney damage^1^. The evaluation of newly diagnosed MM (NDMM), including tumor burden and disease risk stratification, plays a critical role in determining prognosis^2^. The Durie–Salmon (D–S) staging system has been used to approximate the tumor burden since 1975^3^, which demonstrates the correlation between the amount of myeloma and the damage it has caused. It uses the results of blood tests, urine tests, and X-ray radiographs. Conventional radiographs such as magnetic resonance imaging (MRI) and computed tomography (CT) were generally utilized to detect myeloma-associated bone destruction^4^. Advanced techniques such as whole-body (WB) ^18^F-fluorodeoxyglucose (FDG) positron-emission tomography (PET)/CT (FDG-PET) or WB MRI were proposed to yield more accurate results in the D–S PLUS staging system^5^. Compared to MRI, FDG-PET has higher sensitivity for detecting bone marrow infiltration and extramedullary plasmacytomas^6,7^. Hence, FDG-PET has become a routine examination for tumor burden and treatment response evaluation in MM^8–10^. Nevertheless, studies have also reported that ^18^F-FDG PET/CT is not sufficiently sensitive for diffuse disease^11,12^.

Acetate can be utilized by cells to synthesize cholesterol and fatty acids, or oxidized via the tricarboxylic acid cycle to produce energy. Because of this ability, ^11^C-ACT PET is advantageous for evaluating tumors that rely more on fatty acid metabolism than glycolysis, such as prostate cancers, well-differentiated lung cancers, and hepatocellular carcinomas^13–15^. Some researchers have concluded that AC-PET outperformed FDG-PET in the diagnosis, risk stratification, and treatment monitoring of MM. However, the existing studies were limited due to the small number of participants^16–18^, and the correlation between AC-PET imaging analysis and MM prognosis is unclear^19^.

Therefore, the current study aimed to compare the value of AC-PET and FDG-PET in detecting myeloma lesions, evaluating tumor burden, stratifying disease risk, and predicting prognosis in patients with NDMM.

## Materials and methods

### Patients

In this prospective cohort study, NDMM patients who were admitted to the Hematology Department of Peking Union Medical College Hospital, China, between February 2015 and July 2018 were enrolled in this study. The diagnosis and staging of NDMM were based on the International Myeloma Working Group (IMWG) criteria^20^. Patients who had an active infection or were allergic to contrast agents were excluded. The protocol was approved by the Ethics Committee of Peking Union Medical College Hospital. All research was performed in accordance with relevant guidelines and regulations and with the Declaration of Helsinki. All patients provided written informed consent.

### PET-CT evaluation

All patients underwent WB AC-PET and FDG-PET in succession within 1 week after diagnosis without any treatment.

### Tracer synthesis

^11^C-acetate was synthesized following the procedures described by Mitterhauser et al.^21^, using ^11^C-CO_2_ produced by Siemens RDS111 cyclotron as the source of the ^11^C radioisotope. ^18^F-FDG was synthesized on an automated synthesis module (PET (Beijing) Science & Technology Co., Ltd., China). The chemical and radiochemical purities of ^11^C-acetate and ^18^F-FDG were > 95%. Both products were passed through a sterile membrane filter (Millipore filter, 0.22 μm, 25 mm) and cultured for 14 days to ensure no bacteria growing.

### PET imaging

Before PET imaging, a low-dose WB CT scan (120 keV, 100 mAs, 1.3 pitch, 2.5 mm slice thickness, 0.5 s rotation time, 9.0 mGy estimated radiation dose) was obtained for anatomical positioning and attenuation correction. WB AC-PET was then performed using a Polestar m660 time-of-flight PET/CT scanner (SinoUnion Healthcare Inc., China) 20 min after intravenous injection of ^11^C-acetate (555 MBq). At 4 h after AC-PET, the patients received an intravenous injection of ^18^F-FDG (740 MBq), and WB FDG-PET images were acquired 60 min after the injection. PET scanning followed a 2 min/bed position with a 23-slice overlap. Images were reconstructed using an ordered subset expectation maximization algorithm (two iterations, ten subsets, 192 × 192 matrix) and were corrected for CT-based attenuation, dead time, random events, and scatter.

### Image analysis

**Figure 1:**
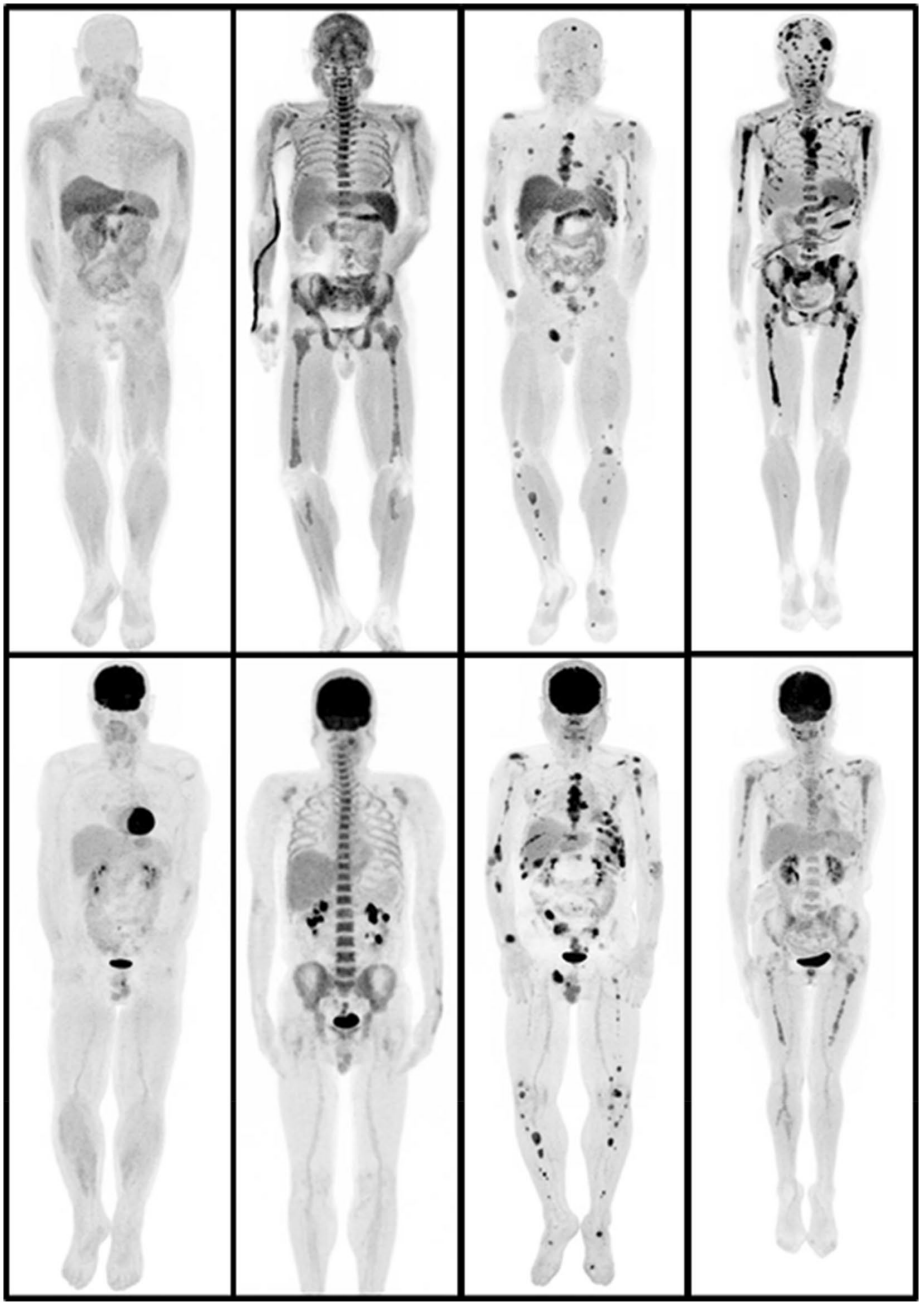
Maximum-intensity-projection images of ^11^C-acetate (upper row) and ^18^F- fluorodeoxyglucose (FDG) (lower row) positron-emission tomography (PET). Bone marrow involvement is shown as negative uptake, diffuse involvement, focal lesions, and diffuse + focal lesions from left to right. (Images by MIM Encore of MIM Software Inc., version number: 6.6.11_buildH213-00, URL: https://www.mimsoftware.com).

The images were reviewed by an experienced expert WJ.Z in nuclear medicine, using MIM Encore software (MIM Software Inc., USA). Any increased uptake that could not be explained by physiological biodistribution was considered abnormal. The bone marrow lesions were further classified into four categories: normal distribution, diffuse uptake, focal uptake, or diffuse + focal uptake (Fig. 1). The classification was subjective. However, it is similar to MR criteria, where five patterns can be described: normal, focal infiltration, diffuse infiltration, combined focal and diffuse infiltration, and “salt-and-pepper” pattern^22^. According to the number of lesions detected, the focal lesions were graded as none (0), oligo (1–9), multiple (10–20), or numerous (> 20). The maximum SUV (SUV_max_) of focal lesions (corrected according to body weight) was recorded. For patients without focal bone marrow lesions, the SUV_max_ of the L5 vertebra was recorded.

### Treatment

The patients received frontline chemotherapy, which mainly included bortezomib, lenalidomide, and thalidomide. Nine patients also received autologous hematopoietic stem cell transplantation (ASCT) following chemotherapy.

### Follow-up

All patients were followed up via outpatient visits or telephone calls once every three months until September 2019. Treatment responses, disease progression, and survival were recorded. The treatment response was assessed according to the IMWG consensus criteria^23^. Progression-free survival (PFS) and overall survival (OS) were defined as the time from the date of diagnosis until the date of disease progression or any cause of death, respectively.

### Data collection

The serum levels of beta 2-microglobulin (β2-MG), lactate dehydrogenase (LDH), M protein according to serum protein electrophoresis, and the 24-h light chain urine level were determined. International staging system (ISS) and D-S staging were determined according to the IMWG criteria^20^. Chromosome aberrations of CD138-sorted bone marrow plasma cells were examined by fluorescence in situ hybridization^24^. High-risk disease was defined as the presence of any of the following: 1q21 amplification, 17p deletion, and t (4; 14) or t (14; 16) translocations.

### Statistical analysis

The detection value and its correlation with tumor burden and the cases of high-risk cytogenetic abnormalities, were compared. Associations between image characteristics and treatment response, PFS, OS were also analyzed. All data were analyzed using SPSS 24.0 software (IBM Corp., Armonk, NY, USA). The chi-square test was used to compare categorical data between AC-PET and FDG-PET. The normality of the data distribution was examined by the Kolmogorov–Smirnov test. Continuous variables with a normal distribution are presented as the mean ± standard deviation (SD), and differences between imaging techniques were assessed using an analysis of variance or an independent-sample *t*-test. Non-normally distributed data are presented as the median (range), and the Mann–Whitney U test was employed to compare the differences between the two groups. Correlations were assessed using the Pearson correlation test or Kendall’s tau-b test. The log-rank test and Breslow test for Kaplan–Meier survival curves was applied for comparisons of PFS and OS. A *P*-value of < 0.05 was deemed statistically significant.

## Results

### Patient characteristics

A total of 70 NDMM patients underwent AC-PET, 64 of whom also underwent FDG-PET. Thus, 64 NDMM patients (41 males and 23 females) were included in the analysis. The patients were aged 36–85 years with a median age of 60 years. Patients’ baseline characteristics are shown in Table 1. Most NDMM patients had advanced disease at the time of diagnosis, with 35 (54.7%) ISS stage III and 38 (59.4%) D–S stage IIIA. Thirty (46.9%) patients were determined to have high-risk diseases.

**Table 1:** Baseline characteristics of the NDMM patients.

Characteristics	n = 64
Gender (male)	41 (64.1%)
Median age (years)	60 (36–85)
**M protein type**	
IgG	28 (43.7%)
IgA	18 (28.1%)
LC	13 (20.3%)
IgD	5 (7.9%)
Hemoglobin (g/L)	97.5 (42–182)
M protein in SPE (g/L)*	35.6 (0–99.8)
24-h urine LC (mg)**	8252 (275–56,000)
BM myeloma cells (%)	26% (0–88%)
LDH > 250 U/L	12 (18.7%)
Serum β2-MG (mg/L)	6.08 (0.6–67.4)
**ISS staging**	
I	9 (14.1%)
II	20 (31.3%)
III	35 (54.7%)
**D–S staging**	
IA	4 (6.3%)
IB	2 (3.1%)
IIA	6 (9.4%)
IIIA	38 (59.4%)
IIIB	14 (21.9%)
**CA by FISH**	
1q21+	24 (37.5%)
RB1−	28 (43.8)
DS319−	28 (43.8%)
17p−	11 (17.2%)
t (14;16)	3 (4.9%)
t (4;14)	7 (10.9%)
t (11;14)	8 (12.5%)
High risk disease	30 (46.9%)

LC, light chain; SPE, serum protein electrophoresis; BM, bone marrow; LDH, lactic dehydrogenase; β2-MG, β2-microglobulin; ISS, International Staging System; D–S, Durie–Salmon; CA, cytogenetic abnormalities; FISH, fluorescence in situ hybridization; *, IgG and IgA only; **, light chain only.

### Comparison of AC-PET and FDG-PET in the detection of NDMM

**Figure 2:**
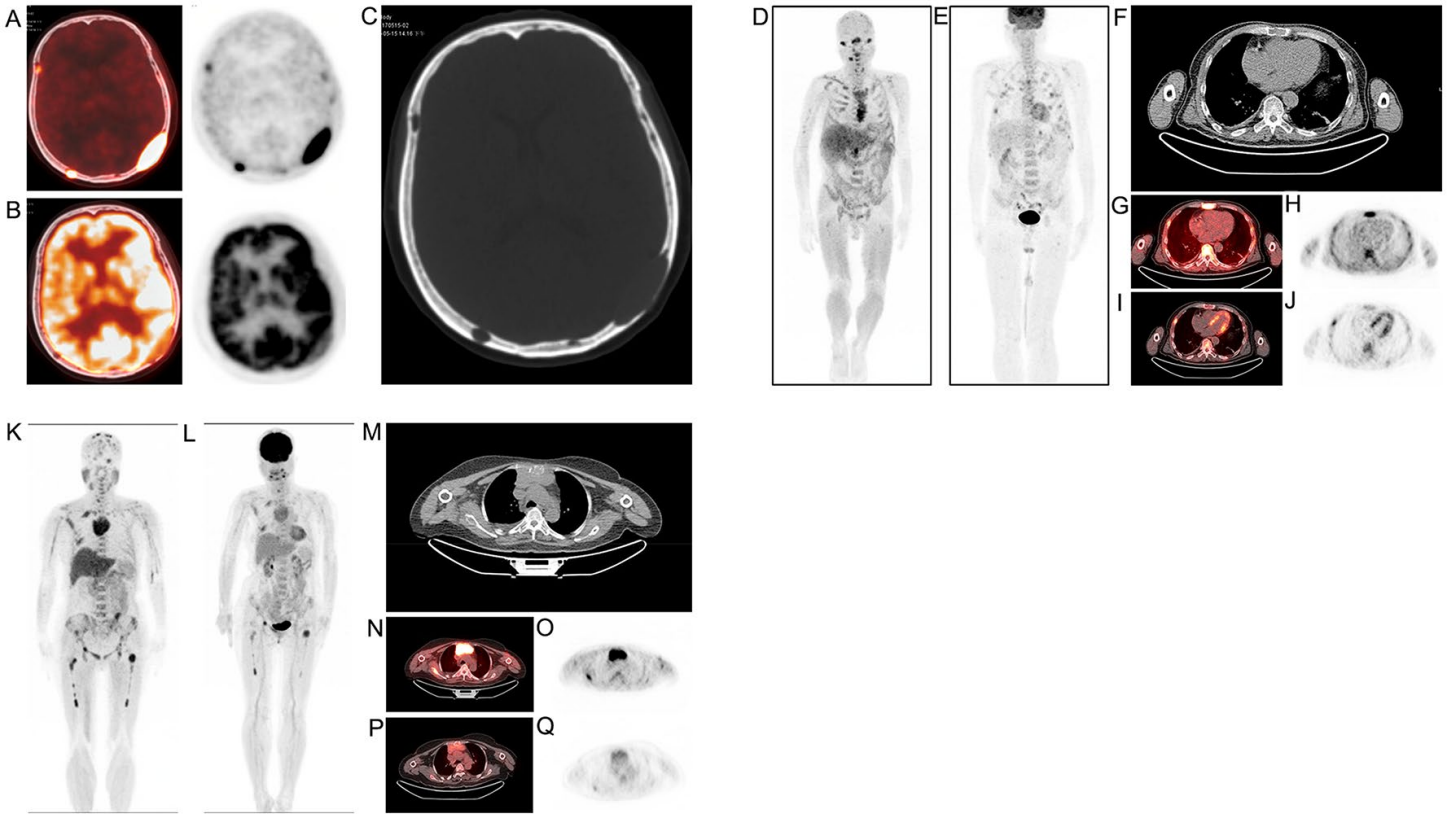
11C-acetate and 18F-FDG PET/CT images. (**A**–**C**) ^11^C-acetate (**A**) and ^18^F-FDG (**B**) PET and CT (**C**) images of the brain of a typical patient, showing multiple osteolytic lesions of the skull. (**D**–**J**) The ^11^C-acetate and ^18^F-FDG PET/CT images in a patient with multiple rib fractures**:** ^11^C-acetate maximum-intensity-projection PET image (**D**); ^18^F-FDG maximum-intensity-projection PET image (**E**); CT image (**F**); ^11^C-acetate PET/CT axial fusion image (**G**); ^11^C-acetate axial PET image (**H**); ^18^F-FDG PET/CT axial fusion image (**I**); ^18^F-FDG axial PET image (**J**). The ^18^F-FDG PET images (**E**, **I**, **J**) show multiple FDG-avid lesions (false positives) of the bilateral ribs, with fracture line shown on the CT image. The ^11^C-acetate PET images (**D**, **G**, **H**) show no focal lesions. (**K**–**Q**) The ^11^C-acetate and ^18^F-FDG PET/CT images of a typical patient with extramedullary disease**:** ^11^C-acetate maximum-intensity-projection PET image (**K**); ^18^F-FDG maximum-intensity-projection PET image (**L**); CT image(**M**); ^11^C-acetate PET/CT axial fusion image (**N**); ^11^C-acetate axial PET image (**O**); ^18^F-FDG PET/CT axial fusion image (**P**); ^18^F-FDG axial PET image (**Q**). Both ^11^C-acetate (**K**) and ^18^F-FDG (**L**) PET images show diffuse and focal involvement of the bone marrow. The CT image shows a mass with soft tissue density posterior to the sternum, which is both ^11^C-acetate- (**N**, **O**) and ^18^F-FDG- (**P**, **Q**) avid. (Images by MIM Encore of MIM Software Inc., version number: 6.6.11_buildH213-00, URL: https://www.mimsoftware.com).

AC-PET showed greater sensitivity than FDG-PET for the detection of MM. The strong physiological uptake by the brain on FDG-PET interfered with the detection of adjacent skull lesions, leading to a high rate of false negatives. In contrast, the background uptake by the brain was very weak on AC-PET; therefore, skull deformation and skull lesions with high SUV were more easily detected by AC-PET (Fig. 2A–C). In addition, FDG-PET has a high rate of false positives at fracture sites. AC-PET was more capable than FDG-PET in distinguishing an old fracture from an active tumor lesion (Fig. 2D–J).

**Figure 3:**
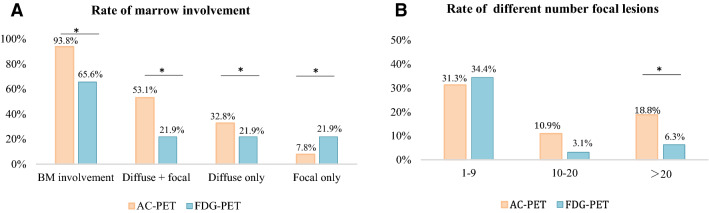
Tumor burden detected by AC-PET and FGD-PET in NDMM patients. (**A**) The positive rate of marrow involvement detected by AC-PET was much higher than by FDG-PET, including diffuse and focal hypermetabolic lesions, diffuse lesions only, and focal lesions only. (**B**) More focal lesions were detected in AC-PET than FDG-PET. BM: bone marrow; the chi-square test was used to compare categorical data between AC-PET and FDG-PET; *P < 0.01.

As shown in Fig. 3, the positive rate of marrow involvement detected by AC-PET was 93.8% (60/64), including 34 patients with both diffuse and focal hypermetabolic lesions, 21 patients with diffuse lesions only, and 5 patients with focal lesions only. The focal lesions showed a median SUV_max_ of 5.68 (range 1.29–47.60) and were mainly located in the pelvis (41.7%), followed by the long bones of the lower limbs (25%), sternum and ribs (20.8%), craniofacial bones (8.3%), and vertebral bones (4.2%). FDG-PET detected marrow involvement in only 65.6% of patients (42/64), a significantly lower rate than that in AC-PET (*P* = 0.004). Of the 42 cases of marrow involvement detected by FDG-PET, 14 were both diffuse and focal hypermetabolic lesions (*P* = 0.001), 14 were diffuse involvement only (*P* = 0.001), and 14 were focal lesions only (*P* = 0.007). In AC-PET, 18.8% of patients (12/64) presented with more than 20 focal lesions, while in FDG-PET, only 6.3% (4/64) were determined to have more than 20 focal lesions (*P* = 0.001).

AC-PET and FDG-PET detected extramedullary plasmacytomas in the same eight patients with similar sensitivity. The images of a typical patient with extramedullary plasmacytomas are shown in Fig. 2K–Q.

### Correlation between PET image characteristics and clinical parameters

**Figure 4:**
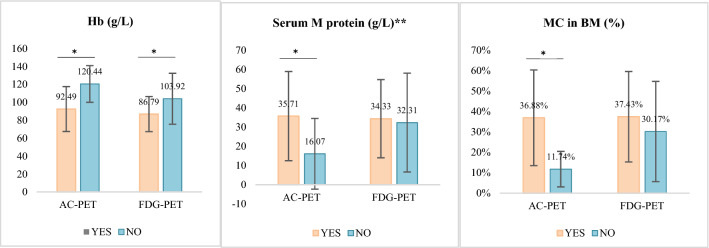
Correlation of fiffuse bone marrow uptake in AC-PET and FDG-PET with tumor burden. YES, patients with diffuse high marrow uptake; No, patients with negative marrow uptake; Hb, hemoglobin; **, IgG and IgA only; MC, myeloma cells; BM, bone marrow; differences of continuous variables with a normal distribution were assessed using independent-sample *t*-test, *P < 0.05.

As indicated in Fig. 4, in the AC-PET group, patients with diffuse high marrow uptake presented with lower hemoglobin (92.49 ± 25.03 vs. 120.44 ± 20.42 g/L, *P* = 0.002), higher M protein in IgG or IgA patients (35.71 ± 23.20 vs. 16.07 ± 18.40 g/L, *P* = 0.05), and a higher percentage of bone marrow plasma cells (36.88 ± 23.44 vs 11.74 ± 8.72%, *P* < 0.001), but with similar calcium and LDH levels (data not shown), compared to patients with negative marrow uptake. In the FDG-PET group, the M protein levels in IgG or IgA subtypes (34.33 ± 20.34 vs. 32.31 ± 25.72 g/L, *P* = 0.778) and myeloma cell densities in the bone marrow (37.43 ± 22.15 vs. 30.17 ± 24.54%, *P* = 0.226) were similar between patients with diffuse high marrow uptake and those with negative uptake. However, a significantly lower hemoglobin level was detected in those with diffuse high bone marrow uptake than those with negative uptake (86.79 ± 19.56 vs. 103.92 ± 28.42 g/L, *P* = 0.008).

Diffuse high marrow uptake in AC-PET was positively correlated with high-risk disease characterized by the presence of high-risk cytogenetic abnormalities (r = 0.29, *P* = 0.020) and the D–S stage (r = 0.39, *P* = 0.001). Moreover, the SUV_max_ of focal lesions in AC-PET was positively correlated with high-risk disease (r = 0.386, *P* = 0.017). Of note, all 12 patients with more than 20 focal lesions on AC-PET were classified as D–S stage III. Diffuse high marrow uptake on FDG-PET was also positively correlated with high-risk disease (r = 0.308, *P* = 0.013) and the D–S stage (r = 0.334, *P* = 0.007). However, the SUV_max_ of focal lesions on FDG-PET showed no correlation with high-risk disease (r = 0.089, *P* = 0.673).

### Comparison of AC-PET and FDG-PET for determining treatment response and clinical outcome

Of the 50 patients who received bortezomib-based regimens, 42 were treated with bortezomib, cyclophosphamide, and dexamethasone; four with bortezomib, lenalidomide, and dexamethasone; two with bortezomib and dexamethasone (BD); and two with BD-cisplatin, doxorubicin, cyclophosphamide, and etoposide (PACE). Of the nine patients who received thalidomide-based regimens, seven were treated with thalidomide, cyclophosphamide, and dexamethasone, and two with thalidomide, dexamethasone, cisplatin, doxorubicin, cyclophosphamide, and etoposide (DT-PACE). The remaining five patients received lenalidomide and dexamethasone. In addition to frontline chemotherapy, nine patients received ASCT following drug treatment. The responses to therapy included 15 cases of stringent complete response, seven cases of complete response, 19 cases of very good partial response, 16 cases of partial response, six cases of stable disease, and one case of progressive disease.

**Figure 5:**
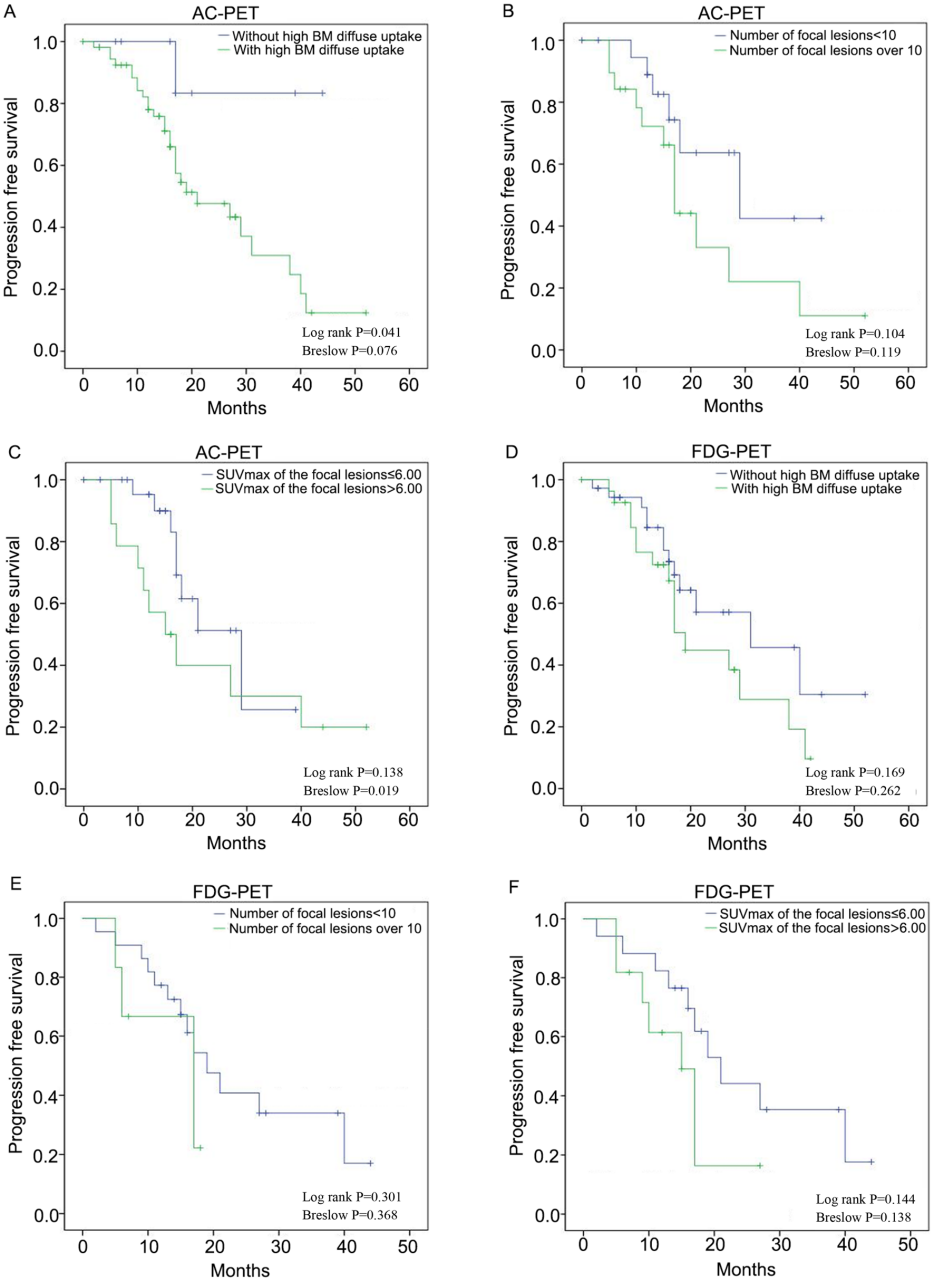
Kaplan–Meier survival analysis of PFS in AC-PET and FDG-PET. (**A**, **D**) Association between PFS and diffuse bone marrow uptake in AC-PET (**A**) and FDG-PET (**D**). (**B**, **E**) Correlation between PFS and the number of focal lesions in AC-PET (**B**) and FDG-PET (**E**). (**C**, **F**) Correlation between PFS and the SUV_max_ of focal lesions in AC-PET (**C**) and FDG-PET (**F**). The log-rank test and Breslow test for Kaplan–Meier survival curves was applied for comparisons of PFS.

Figure 5 and Supplemental Table 1 illustrate that the median follow-up duration was 19 months (range 1–52 months). By the end of the follow-up, 54.7% (35/64) patients remained responsive to therapy, and 35.9% (23/64) patients had died. The estimated 3-year survival rate was 58%. We found no significant correlation between the treatment response and diffuse high bone marrow uptake, the number of focal lesions, and the SUVmax of focal lesions on AC-PET. However, patients with diffuse high marrow uptake on AC-PET had a higher probability of disease relapse (50.9 vs. 11.1%, *P* = 0.033) and a shorter median PFS (21 months, 95% confidence interval [CI] 9.58–32.42 vs. not reached, *P* = 0.041). We also found a positive correlation between disease relapse and the number of focal lesions on AC-PET. The rates of disease relapse in patients with oligo (1–9), multiple (10–20), and numerous (> 20) focal lesions were 30.0, 42.9, and 75.0%, respectively (*P* = 0.046). The median PFS of patients with multiple and numerous focal lesions (17 months, 95% CI 14.7–19.3) on AC-PET was shorter than that of patients with oligo lesions (29 months, 95% CI 9.1–48.9), but the difference was not significant (*P* = 0.104). Compared with patients who had an SUV_max_ of focal lesions of ≤ 6.0 on AC-PET, patients with an SUV_max_ of focal lesions of > 6.0 had a higher rate of disease relapse (71.4 vs 32.0%, *P* = 0.018) and a shorter median PFS (15 months, 95% CI 9.1–20.9 vs. 29 months, 95% CI 15.4–42.7, *P* = 0.017). In contrast, we found no significant correlation between disease relapse or the median PFS and diffuse bone marrow uptake, the number of focal lesions, or the SUV_max_ of focal lesions on FDG-PET (all *P* > 0.05). Kaplan–Meier survival analysis of PFS in AC-PET and FDG-PET showed the presence of diffuse bone marrow uptake and an SUV_max_ of focal lesions of > 6.0 in AC-PET, but not in FDG-PET, predicted a significantly shorter progression-free survival (*P* < 0.05) (Fig. 5).

The OS rate did not significantly differ according to diffuse bone marrow uptake (yes or no), the number of focal lesions (< 10 or ≥ 10), or the SUV_max_ of focal lesions (> 6.0 or ≤ 6.0) on both AC-PET and FDG-PET during the 19 months median follow-up period (all *P* > 0.05).

## Discussion

To the best of our knowledge, this is the largest prospective single-center study to compare dual-tracer PET/CT imaging techniques in NDMM patients. Compared with the more conventional FDG-PET, the novel AC-PET exhibited greater sensitivity for detecting bone marrow infiltration and skull lesions. Diffuse bone marrow uptake on AC-PET was significantly correlated with biomarkers that reflect the tumor burden, and the correlation was stronger than that on FDG-PET. Meanwhile, the SUV_max_ of the focal lesion was significantly correlated with a high-risk disease on AC-PET but not on FDG-PET. Finally, the presence of diffuse bone marrow uptake, more than ten focal lesions, and an SUV_max_ of focal lesions of > 6.0 on AC-PET were significantly associated with disease progression and PFS in this cohort.

The conventional FDG-PET is commonly used to evaluate the baseline tumor burden and therapeutic response^25^. However, it has limited sensitivity in detecting diffuse bone marrow infiltration and focal lesions because of the low uptake of fluorodeoxyglucose in myeloma cells^18^. Several alternate tracers, including ^11^C-acetate, have been investigated to overcome the limitations of FDG-PET. Acetate is a precursor for lipid synthesis. It could be selectively taken up by certain tumor cells that rely more heavily on fatty acid metabolism than on glycolysis^26^. It is known that the energy production of MM predominantly occurs via aerobic glucose metabolism, which includes the tricarboxylic acid cycle^27^, and the production of abnormal immunoglobulins requires active lipid synthesis in plasma cells. Therefore, we observed high lesion uptake but low physiological brain, bone, and bone marrow uptake of the ^11^C-acetate tracer on AC-PET, which also explains why AC-PET was significantly more sensitive than FDG-PET for detecting skull lesions, lesions at bone fracture sites, and bone marrow involvement of NDMM in this study.

There have been some reports comparing AC-PET and FDG-PET in the clinical examination of MM. In two single-case reports, AC-PET appeared to be more sensitive than FDG-PET in assessing the tumor burden and/or response to therapy^16,17^. In addition, there only two cohort studies have been published to date, which compared these two imaging methods for this disease. Ho et al.^19^ reported dual AC-PET and FDG-PET scanning of 35 patients, including 26 with MM, five with smoldering MM (SMM), and four with monoclonal gammopathy of undetermined significances (MGUS). In this cohort study, AC-PET outperformed FDG-PET with a higher sensitivity (84.6 vs. 57.7%) and specificity (100 vs. 93.1%) in distinguishing active MM from SMM or MGUS. In addition, the number of ^11^C-acetate-avid bone lesions was highly correlated with serum β2-MG, and the resolution of ^11^C-acetate marrow activity was correlated with the clinical response to chemotherapy. Another cohort study conducted by Lin et al.^18^ with 15 NDMM patients who underwent both AC-PET and FDG-PET scanning reported that bone marrow ^11^C-acetate uptake in these patients was positively correlated with bone marrow plasma cell infiltration. The study also revealed a significantly higher mean SUV_max_ on AC-PET than on FDG-PET. Our findings verified that AC-PET is more sensitive than FDG-PET for detecting both diffuse and focal MM lesions. Of note, the present study further analyzed the associations between AC-PET image characteristics and clinical outcomes in MM patients. We found diffuse high uptake of bone marrow, more than ten focal lesions, and an SUV_max_ of focal lesions of > 6.00 on AC-PET, but not FDG-PET, were associated with disease progression and PFS. However, such associations were not significant in predicting OS for MM patients. Overall, our results indicate that AC-PET is a sensitive method for evaluating the tumor burden of active MM and is critical for risk stratification as well as disease prognosis, leading to more effective disease intervention and management.

This study had a few limitations. Firstly, this was a single-center observational study with bias inherent to the study’s design. Further, although we have included as many patients as possible in our analysis, the sample size of 64 patients was relatively small. Finally, the median follow-up duration of 19 months was not sufficiently long to allow a thorough analysis of OS. Future multicenter studies with larger sample sizes and longer follow-up durations are needed to confirm the findings of this study.

## Conclusion

In summary, AC-PET outperformed FDG-PET in tumor burden evaluation and risk stratification of NDMM, as well as disease progression prediction. AC-PET is thus a more suitable method for the evaluation of MM.

## Supplementary Information

Supplementary Table 1.

## Data Availability

Data sharing does not apply to this article as no datasets were generated or analyzed during the current study.
