# Supplementary material for: 11C-acetate positron emission tomography is more precise than 18F-fluorodeoxyglucose positron emission tomography in evaluating tumor burden and predicting disease risk of multiple myeloma
Source: Sci Rep. 2021 Nov 12;11:22188. doi: 10.1038/s41598-021-01740-2 (PMC8590058; doi:10.1038/s41598-021-01740-2)
Supplement: Supplementary file 1 — Supplementary Table 1. [file 41598_2021_1740_MOESM1_ESM.doc]

Supplemental Table 1. Association between ^11^C-acetate positron-emission tomography and ^18^F-fluorodeoxyglucose positron-emission tomography according to disease relapse and progression-free survival

|  | Disease relapse,  n (%) | P-value | | | Median PFS,  months (95% CI) | | | P-value | |
| --- | --- | --- | --- | --- | --- | --- | --- | --- | --- |
| Diffuse BM uptake (yes vs. no) |  |  | | |  | | |  | |
| AC-PET n=55 vs. n=9 | 28 (50.9%) vs. 1 (11.1%) | 0.033 | | | 21 (9.58-32.42) vs. NR | | | 0.041 | |
| FDG-PET n=28 vs. n=36 | 16 (57.1%) vs. 13 (36.1%) | 0.094 | | | 19 (16.1-21.9) vs. 31 (12.4-49.7) | | | 0.169 | |
| Focal lesions (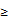10 vs. <10) |  | | |  | | |  | |  |
| AC-PET n=19 vs. n=20 | 12 (63.2%) vs. 6 (30.0%) | | 0.038 | | | 17 (14.7-19.3) vs. 29 (9.1-48.9) | | | 0.104 |
| FDG-PET n=6 vs. n=22 | 4 (66.7%) vs. 13 (59.1%) | | 1.000 | | | 17 (7.7-26.3) vs. 19 (13.2-24.8) | | | 0.301 |
| SUV_max_ >6.0 vs ≤6.0 |  | |  | | |  | | |  |
| AC-PET n=14 vs. n=25 | 10 (71.4%) vs. 8 (32.0%) | | 0.018 | | | 15 (9.1-20.9) vs. 29 (15.4-42.7) | | | 0.017 |
| FDG-PET n=17 vs. n=11 | 10 (58.8%) vs. 7 (63.6%) | | 1.000 | | | 15 (10.0-20.0) vs. 21 (14.9-27.1) | | | 0.144 |

AC-PET, ^11^C-acetate positron-emission tomography; FDG-PET, ^18^F-fluorodeoxyglucose positron-emission tomography; PFS, progression-free survival; BM, bone marrow; CI, confidence interval; NR, not reached; SUV_max_, maximum standardized uptake value
